# The Sequence and a Three-Dimensional Structural Analysis Reveal Substrate Specificity among Snake Venom Phosphodiesterases

**DOI:** 10.3390/toxins11110625

**Published:** 2019-10-28

**Authors:** Anwar Ullah, Kifayat Ullah, Hamid Ali, Christian Betzel, Shafiq ur Rehman

**Affiliations:** 1 Department of Biosciences, COMSATS University Islamabad, Park Road, Tarlai Kalan, Islamabad 45550, Pakistan; kafy.biodiesel@gmail.com (K.U.); hamidpcmd@yahoo.com (H.A.); 2 Institute of Biochemistry and Molecular Biology, University of Hamburg, Laboratory for Structural Biology of Infection and Inflammation, c/o DESY. Build. 22a, Notkestrasse 85, 22607 Hamburg, Germany; 3 Department of Botany, University of Okara, Okara 56300, Punjab, Pakistan; evergreenpk@gmail.com

**Keywords:** snake venom, phosphodiesterases, amino acid sequence and three-dimensional structural analysis, variable substrate specificity, PDE_*Ca* structure–function relationship

## Abstract

(1) Background. Snake venom phosphodiesterases (SVPDEs) are among the least studied venom enzymes. In envenomation, they display various pathological effects, including induction of hypotension, inhibition of platelet aggregation, edema, and paralysis. Until now, there have been no 3D structural studies of these enzymes, thereby preventing structure–function analysis. To enable such investigations, the present work describes the model-based structural and functional characterization of a phosphodiesterase from *Crotalus adamanteus* venom, named PDE_*Ca.* (2) Methods. The PDE_*Ca* structure model was produced and validated using various software (model building: I-TESSER, MODELLER 9v19, Swiss-Model, and validation tools: PROCHECK, ERRAT, Molecular Dynamic Simulation, and Verif3D). (3) Results. The proposed model of the enzyme indicates that the 3D structure of PDE_*Ca* comprises four domains, a somatomedin B domain, a somatomedin B-like domain, an ectonucleotide pyrophosphatase domain, and a DNA/RNA non-specific domain. Sequence and structural analyses suggest that differences in length and composition among homologous snake venom sequences may account for their differences in substrate specificity. Other properties that may influence substrate specificity are the average volume and depth of the active site cavity. (4) Conclusion. Sequence comparisons indicate that SVPDEs exhibit high sequence identity but comparatively low identity with mammalian and bacterial PDEs.

## 1. Introduction

Snake venom is a crude mixture that contains enzymatic and non-enzymatic proteins, peptides, organic compounds of low molecular weight, and inorganic compounds [1,2]. Proteins constitute the major portion (about 90%) of the total dry mass of crude snake venom, with or without catalytic activity, including neurotoxins, cardiotoxins, C-type lectins, proteinases, metalloproteinases, serine proteinases, phospholipases, hyaluronidases, acetylcholinesterases, L-amino acid oxidases, three-finger toxins, phospholipase A_2_s, and nucleases [3,4,5,6,7,8,9]. Metalloproteinases, serine proteinases, phospholipases, and neurotoxins are the most widely studied snake venom proteins, as they occur in high concentrations and are relatively easy to purify [1,2,3,4,5,10,11,12]. Other enzymes, such as nucleases, exist in small quantities and are the least studied. Nucleases are capable of cleaving phosphodiester bonds in nucleic acids, and, in snake venom, they have been classified as endonucleases and exonucleases [13,14]. Phosphodiesterases are generally considered exonucleases [14].

Phosphodiesterases (E.C. No. 3.1.4.1) belong to the Ectonucleotide pyrophosphatase/phosphodiesterase (E-NPP) family of metalloenzymes [13]. Generally, viperid venoms contain more phosphodiesterases (PDEs) than crotalid or elapid venoms [15,16]. Phosphodiesterases cleave phosphodiester bonds in polynucleotides in a sequential manner, starting at the 3′-end, and release 5′-mononucleotides [16]. PDEs have been shown to hydrolyze a wide variety of nucleotides, such as ATP, ADP, NAD^+^, NADP^+^, and GDP [4,17]. Because this enzyme degrades oligonucleotide fragments, there is increasing demand for purified PDE for use in the structural analysis of nucleic acids [17,18].

PDEs have been isolated from various snake venoms, including Deinagkistrodon acutus [19], Bothrops atrox [20], Bothrops alternatus [21], Cerastes vipera [22], Crotalus atrox [23], Crotalus adamanteus [24,25] Crotalus durissus terrificus [25], Protobothrops flavoviridis [26], and Vipera aspis [27]. In addition to snake venoms, they also occur in spider venoms [28]. The structures of human, mouse, and bacterial PDEs have been well studied compared to snake and spider venom PDEs. 

Snake venom PDE was first reported by Uzawa [29]. This is one of the least studied enzymes in snake venom due to the fact that earlier reports showed it to be non-toxic and involved only in digestion [13]. However, recent reports indicate that PDE has a major role in envenomation by hydrolyzing DNA and RNA, releasing adenosine and other purine nucleosides [30,31]. Adenosine induces a variety of pathological and pharmacological effects, such as increased vascular permeability, hypotension, inhibition of platelet aggregation, edema, and paralysis [32,33]. Snake venom phosphodiesterases (SVPDEs) have also been used as therapeutic agents in various diseases and conditions, such as cerebrovascular and cardiovascular diseases, hypertension, and atherosclerosis [34].

SVPDEs are monomeric proteins of high molecular weight (98–140 kDa), with basic pIs (8.4–9.2), and metal cofactors, usually zinc, which are essential for catalytic activity [35,36,37,38]. Some studies report a dimeric structure [13,39,40]. Sometimes, a single venom may contain multiple PDE isoforms [13,37,40].

Although the amino acid sequences of PDEs from various snake species are available in the literature, there is no information regarding their three-dimensional (3D) structures. Therefore, it is difficult to correlate function with structure. In order to enable structure-function studies, here we present a model-based 3D structural characterization of the phosphodiesterase from *Crotalus adamanteus* venom. The PDE_*Ca* structure model was produced and validated using various software (I-TESSER, MODELLER 9v19, Swiss-Model, PROCHECK, ERRAT, and Verif3D). The sequence alignment, structure-based substrate specificity, maturation, and comparison with PDEs from other organisms are also discussed. 

## 2. Results and Discussion

### 2.1. Sequence Alignment Analysis

**Figure 1:**
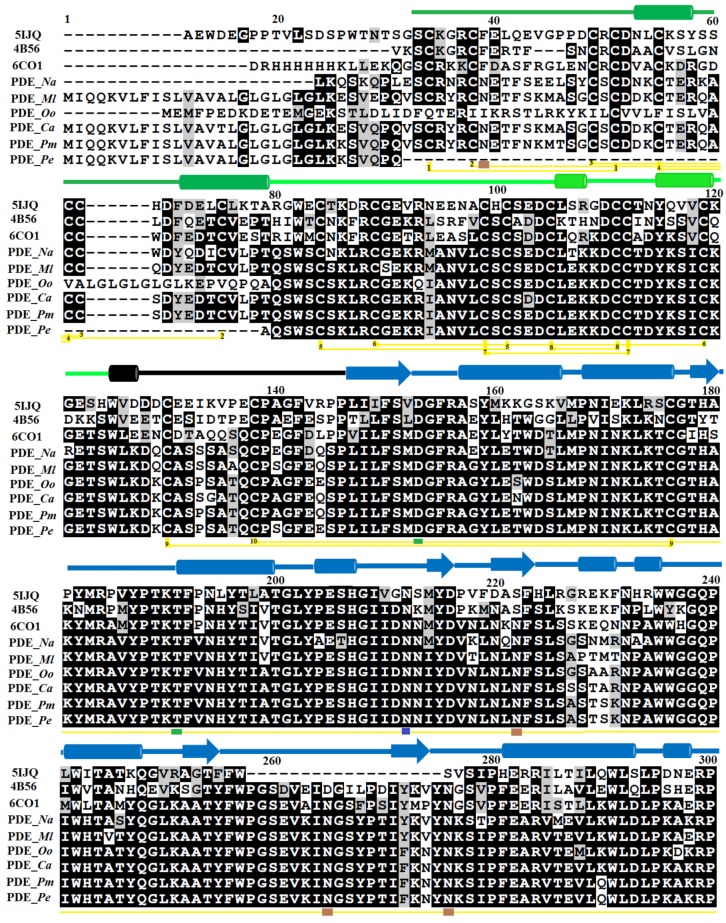

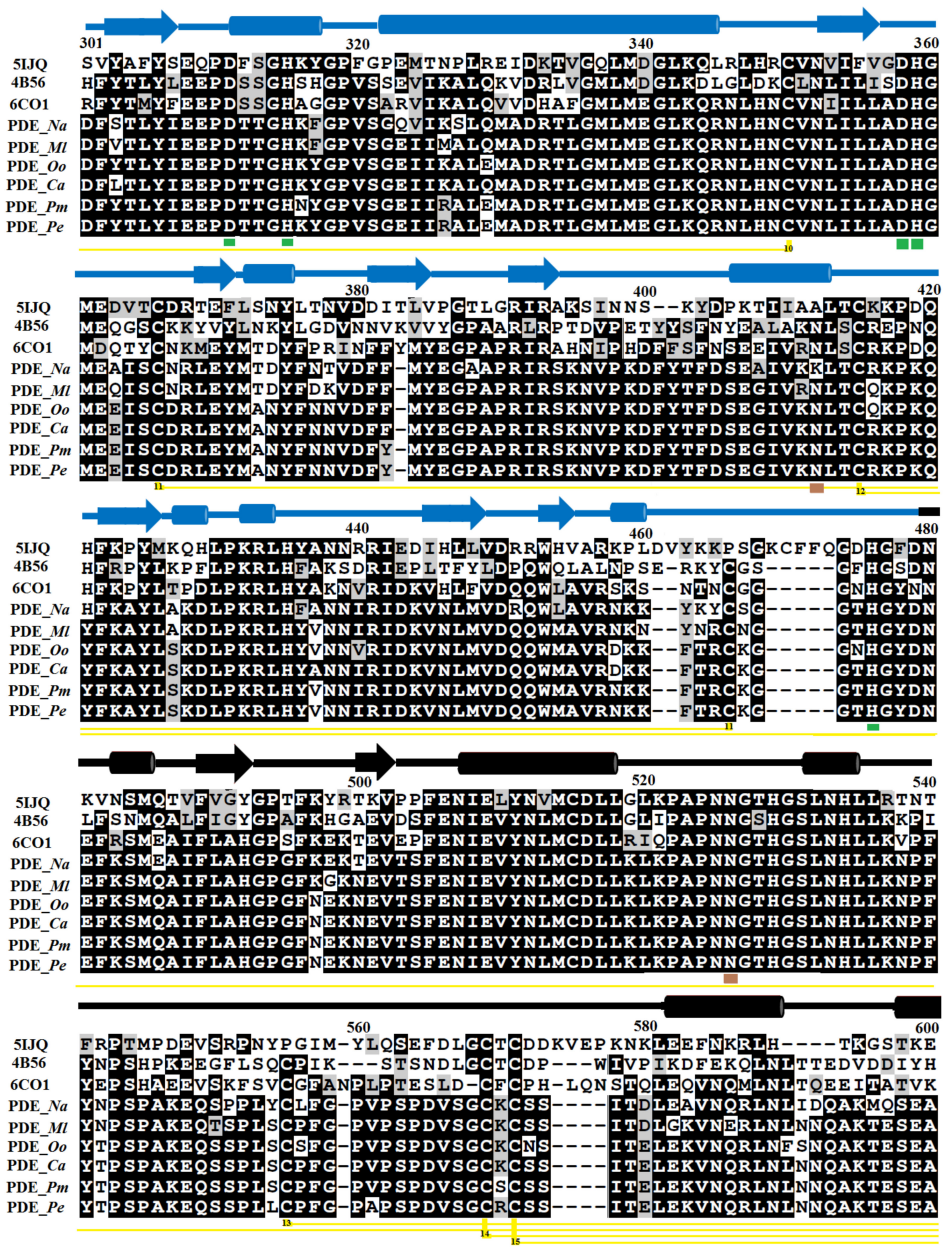

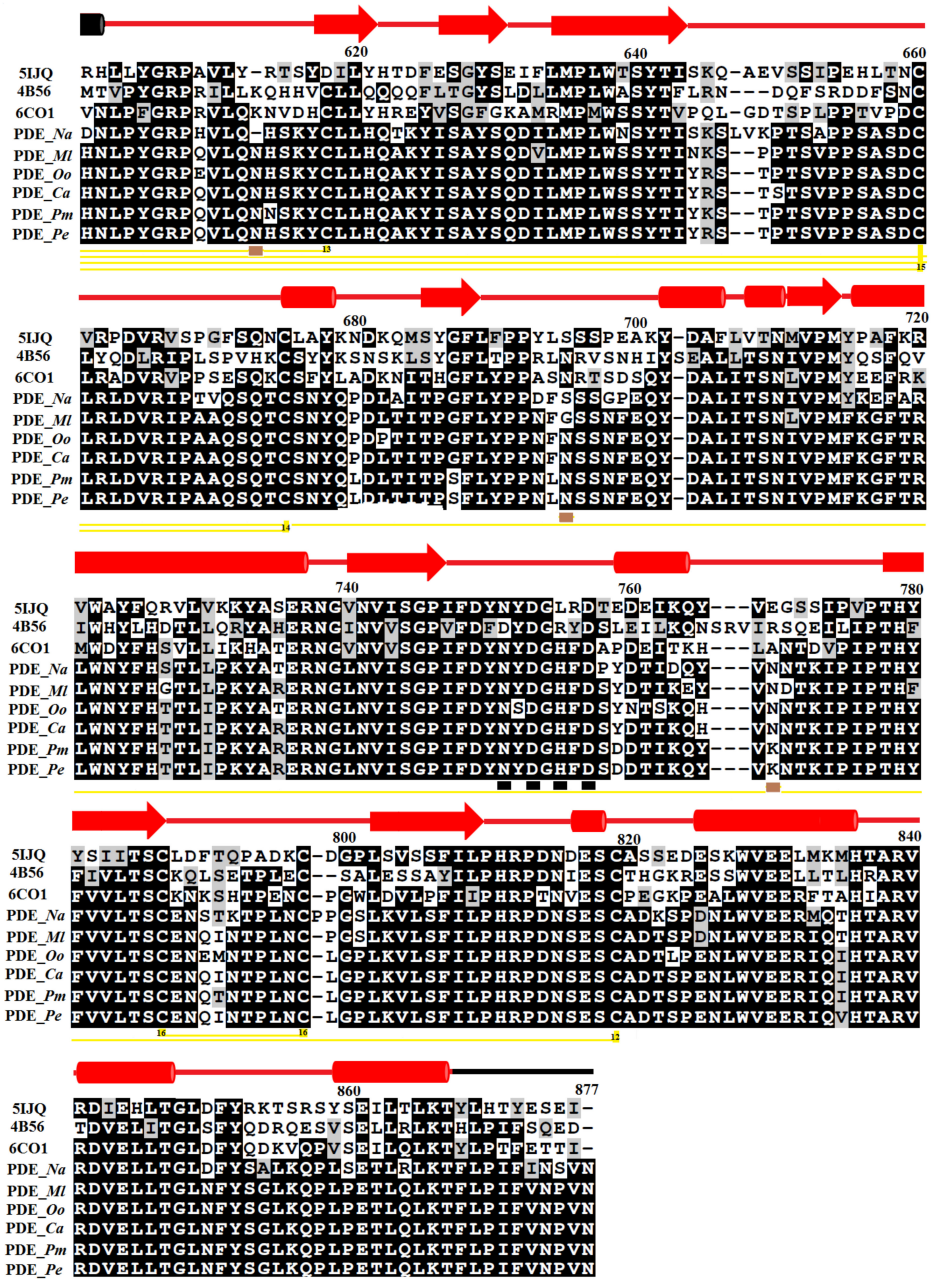
Sequence alignment of snake venom phosphodiesterases (SVPDEs) and mammalian homologs. 5IJQ; crystal of human Autotaxin (ENPP2); 4B56: Ectonucleotide pyrophosphatase phosphodiesterase-1 (NPP1) (*Mus musculus*); 6C01: human Ectonucleotide pyrophosphatase/phosphodiesterase 3 (ENPP3); 5GZ4, phosphodiesterase *Naja atra atra* (Gene Bank ID: A0A2D0TC04), PDE_*Ml*, phosphodiesterase *Macrovipera lebetina* (*Vl*) (Gene Bank ID: AHJ80885.1), PDE_*Oo*, phosphodiesterase *Ovophis okinavensis* (Gene Bank ID: BAN89426.1), PDE_*Ca*, phosphodiesterase *Crotalus adamanteus* (Gene Bank ID: JAS04699.1), PDE_*Pm*, phosphodiesterase *Protobothrops mucrosquamatus* (Gene Bank ID: XP_015675293.1), PDE_*Pe*, phosphodiesterase *Protobothrops elegans* (Gene Bank ID: BAP39928.1). Residues involved in catalysis and metal ion binding are underlined with blue and black, respectively. Cysteine residues that form disulfide bridges are linked (yellow lines). Putatively N-glycosylated amino acid residues are underlined in brown. Amino acid residues in the somatomedin B domain, the somatomedin B-like domain, the ectonucleotide pyrophosphatase/phosphodiesterase domain (also called autotaxin), and the DNA/RNA non-specific domain, are colored green, light green, blue, and red, respectively. Secondary structural elements (alpha helices and beta strands) are shown above the sequence. Sequence numbering corresponds to the PDE_*Ca* precursor protein.

The PDE_*Ca* precursor contains 851 amino acids with 830 amino acid residues in the mature form. Sequence alignment indicates high sequence identity among SVPDEs and comparatively low sequence identity (<65%) with their mammalian counterparts (Table 1). The average sequence identities among SVPDEs and mammalian phosphodiesterases are 90.6% and 58.3%, respectively. The metal ion-binding/active site residues (Zn^+2^ 1 (D153, T191, D358, H359), Zn^+2^ 2 (D311, H315, and H476), and Ca^+2^ (N751, D753, H755, D757) (PDE_*Ca* precursor numbering scheme) are fully conserved among all phosphodiesterases examined, except N751 and H755 in SVPDEs, where these have mutated to D751 and R755, respectively, in mammalian homologs (Figure 1). Amino acid residues around the metal ion binding and active sites are highly conserved among all phosphodiesterases examined. The amino acid sequence of PDE_*Ca* contains 33 cysteine residues, of which 32 form 16 disulfide bridges (Table 2). The generated model and DiANNA web server [38] also confirmed the presence of sixteen disulfide bridges in PDE_*Ca*.

### 2.2. Domain Analysis

The primary structure of mature PDE_*Ca* contains four domains—the somatomedin B domain (residues 33–79), somatomedin B-like domain (residues 81–124), ectonucleotide pyrophosphatase/phosphodiesterase domain (also called autotaxin) (residues 147–479), and the DNA/RNA non-specific domain (residues 534–867) (Figure 1). The regions of amino acid residues from 124–146, 480–533, and 868–877 are connecting segments.

**Figure 2:**
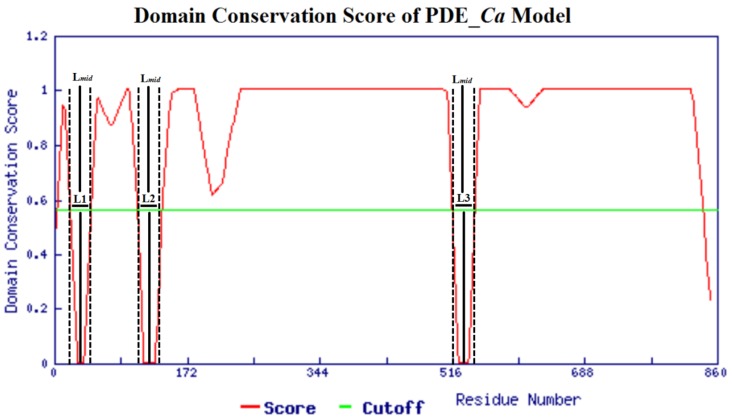
The ThreaDom-based domain prediction in the domain conservation score profile. Four domains are separated by three connecting segments. Vertical dotted lines indicate the start and end locations of each putative connecting segment. Vertical solid lines denote predicted boundaries at the middle of the connecting segments.

The ThreaDom (Threading-based Protein Domain Prediction) online web server [41] for domain conservation indicated that the domain architecture of PDE_*Ca* is fully conserved, with other proteins in the Protein Data Bank (PDB) containing a similar structure fold (Figure 2). The molecular weights of PDE_*Ca* in zymogen and the mature forms were 96.37 and 93.10 kDa, with corresponding pIs of 8.13 and 8.05, respectively [42]. The theoretically calculated molecular weights and pI*s* accord with the experimentally measured values for other SVPDEs [43,44,45,46,47].

### 2.3. Glycosylation Sites

The primary structure of PDE_*Ca* contains 60 asparagine (N) residues (Protparam, [48]), of which nine were identified as potential glycosylation sites using the NetNGlyc 1.0 Server (N39, N222, N265, N276, N412, N526, N613, N695, and N771) (Figure 1). These asparagine residues are fully conserved among SVPDEs (Figure 1). Glycosylation sites were also confirmed with the Scan Prosite tool [49]. The primary structure of *Vipera lebetina* has also been shown to contain nine putative *N*-glycosylation sites [43]. PDEs of *B. jararaca* and *Walterinnesia aegyptia* contain 33% and 24% carbohydrates, respectively [48,49].

### 2.4. Homology Modeling and Model Evaluation

The homology model for three-dimensional (3D) structure characterization was produced using various online modeling servers (I-TESSER [50], MODELLER 9v19 program [51], and SWISS Model [52], using atomic coordinates of human ectonucleotide pyrophosphatase/phosphodiesterase 3 (PDB ID: 6C01, amino acid sequence identity 63.39%) as a template [53]. The best model was chosen based on analyses using the PROCHECK, ERRAT, and Verif3D software [54,55,56,57].

**Figure 3:**
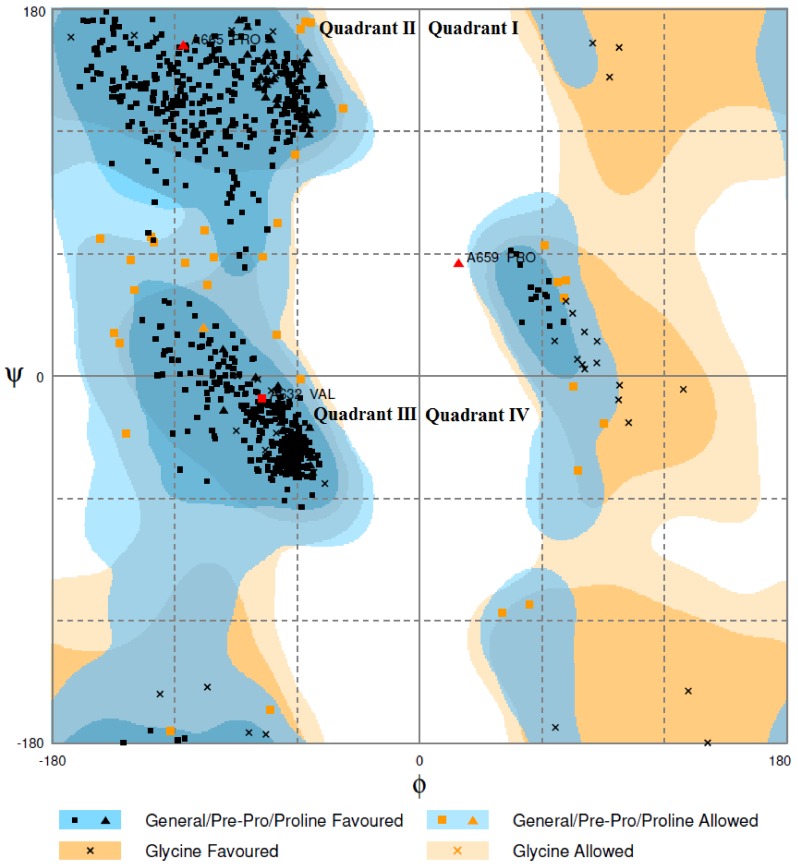
A Ramachandran plot of the modeled structure of PDE_*Ca*. In total, >95% of the amino acid residues are in the most favored region, while the remaining 5% are in the allowed region with no residues in the forbidden/disallowed region. Quadrant I displays a region where multiple conformations are allowed. Quadrant II shows the biggest region in the graph, with the most favorable conformations of atoms. Quadrant III shows the next biggest region in the graph, where the right-handed alpha helices lie. Quadrant IV has almost no outlined region. This conformation is disfavored due to steric clash.

The PROCHECK analysis of the best model shows that >95% of the amino acid residues are in the most favored region of the graph (Figure 3). The remaining 5% are in the allowed region with no residues in the forbidden/disallowed region. ERRAT analysis shows an overall quality factor of 87.88 for the PDE_*Ca* model, which lies in the average quality range for 3D protein structures [55].

### 2.5. Molecular Dynamics Simulation

GROMACS, AMBER16, MDWeb, and MDMobby [58,59,60] all produced the same results. MD simulation analysis indicated that all structural parameters, such as chirality, disulfide bonds, and the absence of steric clashes, were correct (Figure S1A). The two basic assessments (root mean square deviation (RMSD) and radius of gyration (RG)) used to validate the structures through MD simulation were analyzed. The RMSD deviation indicated that the PDE_*Ca* structure did not deviate more than 1 Å from the initial structure, and the RG was also maintained at around 31.7 Å (Figure S1B,C). Flexibility analysis (Bfactor and RMSD per residue), identified some flexible regions, located mostly in loop regions (Figure S1C,D). All these analyses indicate that the simulated structure does not exhibit critical structural deformations.

### 2.6. Overall Structure of PDE_Ca

**Figure 4:**
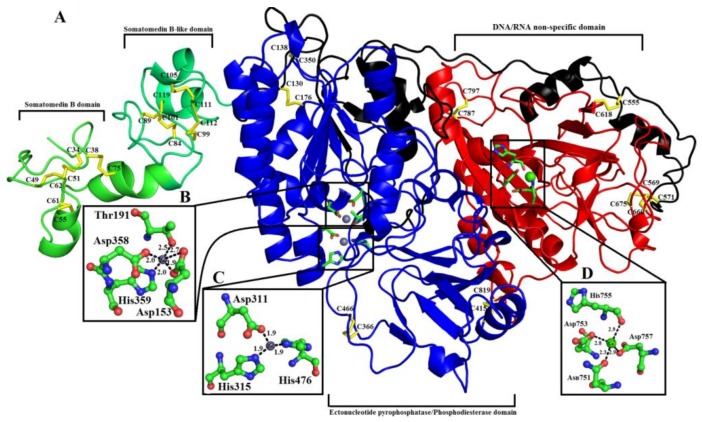
Overall structure of PDE_*Ca*: (**A**) cartoon representation. The active site and metal ion-binding residues are shown as green sticks. Zn^+2^ and Ca^+2^ ions are shown as gray and green spheres, respectively. Disulfide bridges are represented by yellow sticks. (**B**–**D**), residues involved in Zn^+2^ ion-binding, catalysis, and Ca^+2^ ion-binding are highlighted. Parts of the secondary structure belonging to the somatomedin B domain, somatomedin B-like domain, ectonucleotide pyrophosphatase/phosphodiesterase domain, DNA/RNA non-specific domain, and connecting are colored in green, light green, blue, red, and black, respectively.

The 3D structure of PDE_*Ca* is similar to that of the other members of the alkaline phosphatase-like superfamily (ALP-like superfamily) [25,61,62,63]. PDE_*Ca* has a complex structure. It is a multi-domain protein that consists of four domains—a somatomedin B domain, a somatomedin B-like domain, an ectonucleotide pyrophosphatase/phosphodiesterase domain (also called autotaxin), and a DNA/RNA non-specific domain (Figure 4). These domains are briefly described below.

#### 2.6.1. Somatomedin B Domain

**Figure 5:**
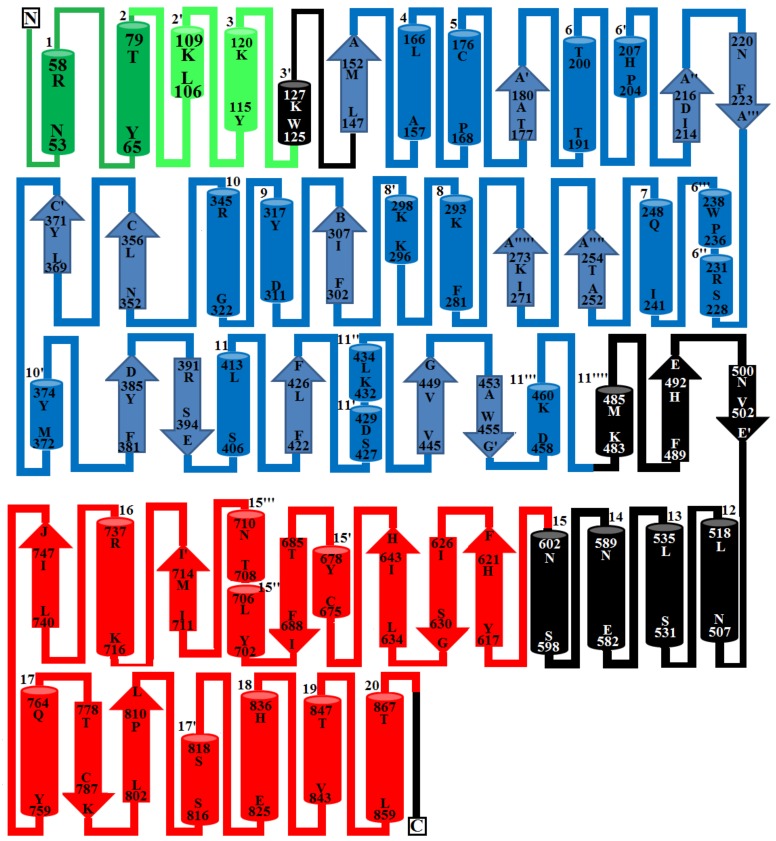
Topology diagram of PDE_*Ca*. The alpha helices (1–20) and beta strands (A–L) are represented as cylinders and arrows, respectively. Short alpha helices and beta strands are shown as primes (e.g., 17’). Secondary structures and amino acid residues in alpha helices and beta strands were assigned from the primary sequence using the program DSSP [66] and were confirmed with PyMOL from the tertiary structure. Parts of the secondary structure belonging to the somatomedin B, somatomedin B-like, ectonucleotide pyrophosphatase/phosphodiesterase, and DNA/RNA non-specific domains, as well as the connecting segments (UN), are colored in green, light green, blue, red, and black, respectively.

The somatomedin B domain (SMB) is located at the N-terminus of the protein and comprises amino acid residues 33–79 (Figure 1, Figure 4 and Figure 5). It has two alpha helices (Figure 4 and Figure 5). The SMB domain is stabilized by four intrachain disulfide bridges, one salt bridge (between Asp52-Arg58) (Table 3), and extensive hydrogen bonding (14 intrachain H-bonds).

#### 2.6.2. Somatomedin B-like Domain

The somatomedin B domain connects to another domain called the Somatomedin B-like domain (SMB-like). This domain consists of residues 81 to 124 (Figure 1). Like the somatomedin B domain, it also contains eight cysteine residues in four disulfide bridges (Figure 1). The secondary structure of this domain contains two alpha helices (Figure 4 and Figure 5). Beside its disulfides, the SMB-like domain is stabilized by four salt bridges (Arg82-Glu85, Arg87-Asp104, Arg87-Asp98, Lys102-Asp98), and thirteen interchain H-bonds (four with the SMB domain and nine with the PDE domain), as well as 24 intrachain H-bonds.

Both the SMB and SMB-like domains are highly compact. Disulfide bridges are arranged in the centers of both domains, forming covalently bonded cores (Figure 4).

#### 2.6.3. Ectonucleotide Pyrophosphatase/Phosphodiesterase Domain

The SMB-like domain connects to the catalytic domain, called the ectonucleotide pyrophosphatase/phosphodiesterase (ENPP/PDE) domain, through a short connecting segment of 22 amino acid residues (125–146) (Figure 1, Figure 4 and Figure 5).

**Figure 6:**
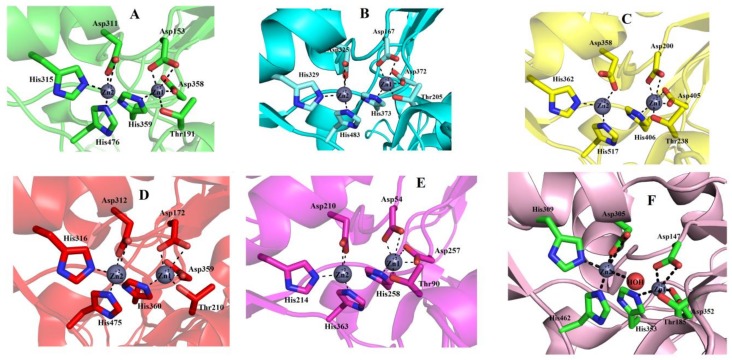
Active site comparison of (**A**) PDE_*Ca* with (**B**) human Ectonucleotide pyrophosphatase/phosphodiesterase 3 (ENPP3), (**C**) Ectonucleotide pyrophosphatase-phosphodiesterase-1 (NPP1) (*Mus musculus*), (**D**) ENPP2 (human Autotaxin), (**E**) Xac Nucleotide Pyrophosphatase/Phosphodiesterase (*Xanthomonas axonopodis*), and (F) Phosphodiesterase *Naja atra*. Metal ion-binding amino acid residues are displayed as sticks and metal ions as grey spheres.

The ENPP/PDE domain consists of amino acid residues 147–479 (Figure 1). It contains five cysteine residues, two of which make an intrachain disulfide bridge and three of which make interchain disulfide bridges (two with the connecting segment and one with the DNA/RNA non-specific domain) (Figure 1 and Figure 4) (Table 2). This domain also contains the active site residues, together with the two zinc ions (Figure 4). One of the zinc ions (Zn^+2^1) is coordinated by four amino acids (Asp153, Thr191, Asp358, and His59), and the other one (Zn^+2^2) is coordinated by three residues (Asp311, His315, and His476). All these catalytic amino acid residues are fully conserved in mouse NPP1 [62], human NPP3 (61), human autotaxin ENPP2 [64], and bacterial PDE [29] (Figure 6).

The secondary structure of this domain contains 14 beta strands and 14 alpha helices (Figure 5). Of the fourteen alpha helices and beta strands, seven and five are short alpha helices and beta strands, respectively. This domain is stabilized by interchain disulfide bridges (one with the SMB-like domain and seven with the DNA/RNA non-specific domain), 13 salt bridges (Table 3), and numerous interchain H-bonds.

#### 2.6.4. DNA/RNA Non-Specific Domain

This domain comprises residues 603 to 867 (Figure 1). It contains nine cysteines that form one intrachain and three interchain disulfides (one with the PDE domain and two with the connecting segment) (Figure 4). This domain also contains the Ca^2+^-binding loop (Figure 4). The secondary structure of this domain contains seven beta strands and ten alpha helices (Figure 4 and Figure 5). This domain is connected to the PDE domains through a long loop, called the lasso loop [65]. This domain is stabilized by four disulfides, eight salt bridges, and numerous H-bonds.

#### 2.6.5. Metal Ion-Binding Sites

SVPDEs are metalloenzymes that contain zinc and calcium ions [6,26,35,36,67]. The zinc ion participates in the active site and is important for catalytic activity [26,35,36,37,38,39,40,41,42,43,44,45,46,47,48,49,50,51,52,53,54,55,56,57,58,59,60,61,62,63,64,65,66,67]. In the modeled structure of PDE_*Ca*, two zinc ions and one calcium ion were found (Figure 4). Of the two zinc ions, one (Zn^+2^1) is coordinated by amino acid residues Asp153, Thr191, His359, and Asp358 (Figure 4), and the other (Zn^+2^2) is coordinated by amino acid residues Asp311, His315, and His476 (Figure 4). The Ca^+2^ is coordinated by amino acid residues Asn751, Asp753, His755, and Asp757 (Figure 4). The metal ion comparison with the human Ectonucleotide pyrophosphatase/phosphodiesterase 3, Ectonucleotide pyrophosphatase-phosphodiesterase-1 (*Mus musculus*), ENPP2 (Human Autotaxin), Xac Nucleotide Pyrophosphatase/Phosphodiesterase (*Xanthomonas axonopodis*), and Taiwan cobra (*Naja atra atra*) PDE (PDB ID: 5GZ4 and 5GZ5) shows that the amino acid residues coordinating these metal ions are fully conserved (Figure 1A–F and Figure 6A–F).

### 2.7. Structural Basis for Substrate Specificity of Snake Venom Phosphodiesterases

For substrates other than oligonucleotides, SVPDEs display variable substrate specificity [43,44,45,46,47]. Among the SVPDEs for which substrate specificity has been studied, the PDEs from *Vipera lebetina* and *Daboia russelli russelli* hydrolyze ADP [43], while PDEs from *Crotalus adamanteus, Trimeresurus stejnegeri* and *Bothrops jararaca* hydrolyze ATP [45,46,47].

**Figure 7:**
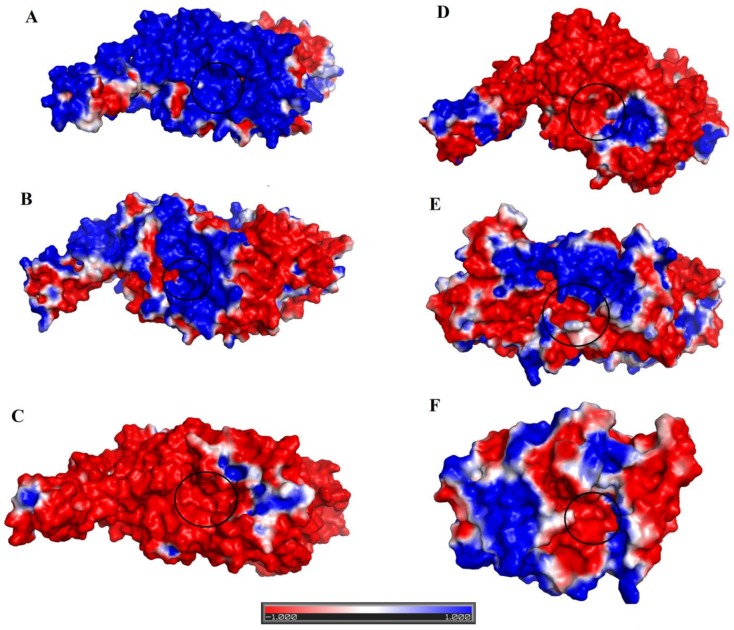
Surface charge distributions of (**A**) PDE_*Ca,* (**B**) PDE_*Vl,* (C) human Ectonucleotide pyrophosphatase/phosphodiesterase 3 (ENPP3), (**D**) Ectonucleotide pyrophosphatase- phosphodiesterase-1 (NPP1) (*Mus musculus*), (**E**) ENPP2 (human Autotaxin), and (**F**) Xac Nucleotide Pyrophosphatase/Phosphodiesterase (*Xanthomonas axonopodis*). Blue, red, and white represent the positive, negative, and neutral regions, respectively. Black circles indicate the location of the active-site pocket.

To explain these broad specificities of SVPDEs, the structures of two other PDEs from *Vipera lebetina* (PDE_*Vl*) and *Bothrops atrox* (PDE_*Ba*) were modeled, using the same modeling and validation programs used for the PDE_*Ca* structural model. The atomic coordinate of human Ectonucleotide pyrophosphatase/phosphodiesterase 3 (PDB ID: 6C01, amino acid sequence identity 64.09% and 62.91% with PDE_*Vl and* PDE_*Ba,* respectively) was used as a template. The Ramachandran plot analysis indicates that in both the modeled structures, 98% of amino acid residues were in the favored region of the plot, and 2% were in the allowed region (Figures S2 and S3). The ERRAT analysis shows an overall quality factor of 90 for the modeled structure, which lies in the average quality range for the protein 3D structures (Figures S4 and S5) [55]. The modeled structures of the PDE_*Ca*, PDE of *Vipera lebetina* (PDE_*Vl*), and *Bothrops atrox* (PDE_*Ba*) were compared, taking into account the active site residue composition, active site cavity volume and average depth, and the surface charge distribution (Figure 7, Table 4). The active site’s amino acid residues are the same for these enzymes. However, the average active site’s cavity volume, the average depth of the active site, and the surface charge distribution vary considerably (Figure 7, Table 4).

The average active site cavity volumes of PDE_*Ca*, PDE_*Vl*, and PDE_*Ba* are 6608.25, 3985.03, and 2243.11 Å^3^, respectively, with corresponding average depths of 21.39, 12.54, and 9.86 Å, respectively (Table 4). These values indicate that the average active site cavity volume and depth of PDE_*Ca* is much larger than that of either PDE_*Vl* or PDE_*Ba*. These characteristics permit larger substrates (ATP) to access the active site of PDE_*Ca*, while preventing it for PDE_*Vl*.

Another factor that affects the substrate specificity of these enzymes is the surface charge distribution (Figure 7). The surface charge of PDE_*Ca* (overall and around the active site) is highly positive (Figure 7A), for PDE_*Vl* it is partially positive and negative (Figure 7B). Analysis of the active site cavity volume and its average depth indicate that SVPDEs with small active site cavity volumes and average depths (like *Vipera lebetina, Daboia russelli russelli,* and *Cerastes cerastes* ) (Table 4) show a high preference for ADP, while other SVPDEs with large active site volumes and average depths (like PDEs from *Crotalus adamanteus, Trimeresurus stejnegeri,* and *Bothrops jararaca*) show a high preference for ATP.

### 2.8. Structural Alignment between PDE_Ca, Human ENPP3, Mouse NPP1, Human Autotaxin, Xa NPP1, PDE_Vl, PDE_Ba and Naja atra atra PDE.

**Figure 8:**
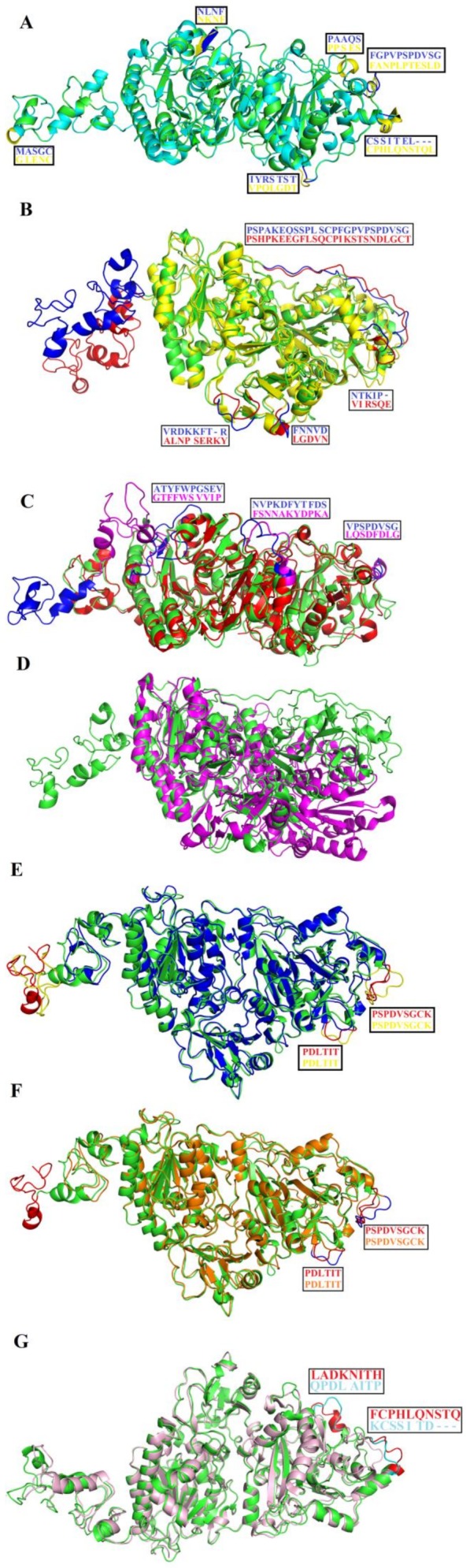
Structural alignment among SVPDEs (PDE_*Ca*, PDE_*Vl,* and PDE_*Ba*) and their mammalian and bacterial counterparts; (**A**) PDE_*Ca* (green) align human Ectonucleotide pyrophosphatase/phosphodiesterase 3 (ENPP3) (Cyan), (**B**) PDE_*Ca* (green) align Ectonucleotide pyrophosphatase-phosphodiesterase-1 (NPP1) (*Mus musculus*) (yellow), (**C**) PDE_*Ca* (green) align ENPP2 (human Autotaxin) (red), (**D**) PDE_*Ca* (green) align Xac Nucleotide Pyrophosphatase/Phosphodiesterase (*Xanthomonas axonopodis*) (magenta), (**E**) PDE_*Ca* (green) align *Vipera lebetina* phosphodiesterase (blue), (**F**) PDE_*Ca* (green) align *Bothrops atrox* phosphodiesterase (orange), **(G)** PDE_*Ca* (green) align 5ZG4 (light pink). Loops exhibiting differences and their corresponding amino acid residues are shown in boxes and colored in blue, red, magenta, orange, yellow, and cyan for PDE_*Ca,* ENPP3, NPP1, PDE_*Vl,* PDE_*Ba,* and 5GZ4, respectively.

The structural alignment between PDE_*Ca*, human ENPP3 [53], mouse NPP1 [61], human autotaxin [63], Xa NPP1 [62], phosphodiesterase from *Vipera lebetina* (PDE_*Vl*), *Bothrops atrox* (PDE_*Ba*), and the PDE from Taiwan cobra (*Naja atra atra*; PDB ID: 5GZ4 and 5GZ5) shows that the three-dimensional structural folds of these enzymes are similar and that all of them align well, with an RMSD value range between 0.21 and 0.92 (average RMSD value of 0.61) (Table 5) (Figure 8). They have the same active site residues (Figure 1 and Figure 6) and disulfide bridges (Figure 1). However, the amino acid residues in the loop regions vary considerably, both in composition and length (Figure 8). For this reason, the surface charge distribution also varies (Figure 7A–G), which may impart variable substrate specificity to these enzymes [45,68,69]. The overall surface charge for the SVPDEs is positive, while it is negative for human ENPP3, mouse NPP1, human autotaxin, and Xa NPP1 (Figure 7C–F). The average active site cavity volume and average depth also vary among these enzymes (Table 4).

The phylogenetic tree analysis indicates that PDE_*Ca* has a close evolutionary relationship with SVPDEs and PDEs from human beings and mice (Figure S6).

### 2.9. Maturation Mechanism for SVPDEs

**Figure 9:**
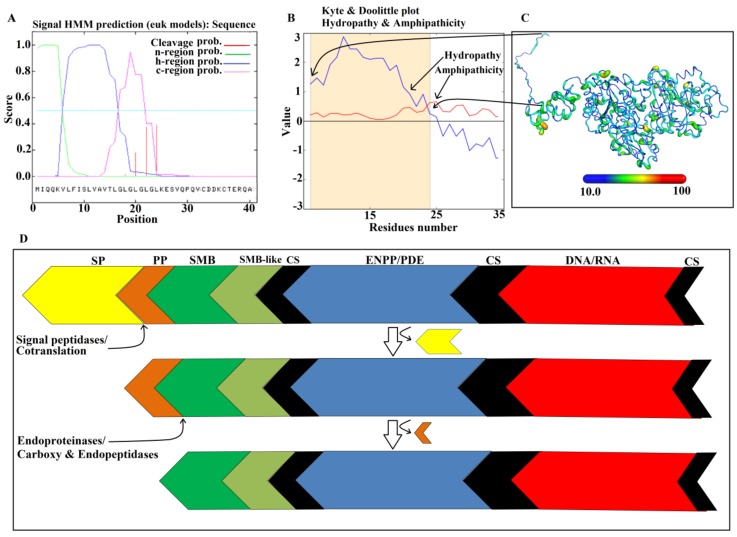
Maturation/processing mechanism for PDE_*Ca.* (**A**) A signalP-HMM prediction plot for PDE_*Ca.* (**B**) A Kyte and Doolittle plot for signal and activation peptides. (**C**) Ribbon representation of PDE_*Ca* colored by B-factor (temperature). (**D**) The prepropeptide of PDE_*Ca* with the signal peptide (colored in yellow), activation peptide (colored in brown), and the mature protein with four domains colored in green (somatomedin B domain), light green (somatomedin B-like domain), blue (Ectonucleotide pyrophosphatase/Phosphodiesterase domain), and red (DNA/RNA non-specific domain). The connecting segments are colored in black.

PDE_*Ca*, like other SVPDEs, is synthesized as a precursor protein (zymogen) [43,45]. The immature PDE_*Ca* contains 851 amino acid residues [64] (Figure 1), in which the first 23 amino acid residues belong to a signal peptide (confirmed with SignalP 3.0 [70], Figure 9A), eight amino acid residues to the activation peptide, and the remaining 820 amino acid residues to the mature protein [48]. The signal peptide prevents the protein from proper folding and is removed cotranslationally or by signal peptidases [71,72]. The Kyte and Doolittle hydropathy plot [42] indicates that this region is located in the hydrophilic part (Figure 9B). The function of the activation peptide is unknown. However, this is considered important for proper folding of the protein, as described for spider venom and plant proteins [2,73]. This part is removed by endopeptidases [74] (Figure 9D). It is also located in the hydrophilic region of the Kyte and Doolittle hydropathy plot [42], and it is exposed on the surface of the protein (and thereby accessible to peptidases). The remaining peptide does not undergo further processing.

## 3. Materials and Methods

### 3.1. Sequence Retrieval and Multiple Sequence Alignment

The amino acid sequence of PDE_*Ca* (851 amino acid residues) (gene bank accession no. JAS04699.1; UniProt ID: A0A0F7Z2Q3) [64] was obtained from the NCBI (National Centre for Biotechnology Information) protein database (http://www.ncbi.nlm.nih.gov/protein). The signal peptide was identified using the SignalP 3.0 server [70] with default parameters. The amino acid sequence of PD_*Ca* was used as a query for searching homologous proteins from the non-redundant database by searching with the NCBI Protein BLAST using default parameters. The multiple sequence alignment of the selected homologous protein sequences, including the amino acid sequence of PD_*Ca,* was generated using MUSCLE [75]. The aligned sequences were edited and colored in Box-shade V3.21 [76].

### 3.2. Sequence Logo Generated from Multiple Sequence Alignment

The Weblogo 3.2 [77,78] was used to illustrate the conservation patterns of amino acids in the protein sequence, and graphically represent the multiple sequence alignment, using default parameters, except for the composition, which was done without adjustment.

### 3.3. In Silico Analysis of the Domain and Biochemical Properties of the PDE_Ca

The PDE_*Ca* primary sequence was analyzed for the presence of domains/motifs using the conserved domain search tool [79], available at http://www.ncbi.nlm.nih.gov/Structure/cdd/wrpsb.cgi. The protparam and Compute pI/MW tools from the ExPASy Proteomics server (http://web.expasy.org/compute_pi/) [42] were used to compute the isoelectric point (p*I*) and molecular weight of the protein.

### 3.4. Prediction of Ligand Binding and Glycosylation Sites

The 3DLigandSite-Ligand binding site prediction Server [80] was used for ligand binding amino acid residues in PDE_*Ca,* while putative glycosylation sites were predicted using the NetNGlyc 1.0 Server [48] and ScanProsite tool [81], with default parameters.

### 3.5. Disulfide Bond Prediction

The DiANNA web server [38] and Dinosolve [82,83,84,85,86] were used for prediction of disulfide bridges in PDE_*Ca*.

### 3.6. Homology Model Building of PDE_Ca

The 3D model of PDE_*Ca* was generated using various online proteins modeling programs, such as I-TESSER [87], the MODELLER 9v19 program [51], and SWISS Model [52], using the atomic coordinates of human Ectonucleotide pyrophosphatase/phosphodiesterase 3 (PDB ID: 6C01, amino acid sequence identity 63.39%) as a template [53]. The final model was selected based on the quality and validation reports generated by PROCHECK [50].

### 3.7. Molecular Dynamics Simulation

The modeled structure of PDE_*Ca* was validated through MD simulation using various programs, like AMBER16 [58], GROMACS [59], MDweb, and MDMoby [60]. The all-atom protein interaction was determined using the FF14SB force field [85]. The web-server H^++^ [84] was used to determine the protonation states of the amino acid side chain at pH 7.0. Chloride ions were used for system neutralization and were placed in a rectangular box of TIP3P water and extended to at least 15 Å from any protein atom. For the removal of bad contacts from the structure, the system was energy minimized for 500 conjugate gradients steps by applying a constant force constraint of 15 kcal/mol. Å^2^. The system was then heated gradually from 0 to 300 K for 250 ps with a constant atom number, volume, and temperature (NVT) ensemble, at the same time that the protein was restrained with a constant force of 10 kcal/mol. Å^2^. The equilibration step was carried out using the constant atom number, pressure, and temperature (NPT) ensemble for 500 ps, and the simulation was done for 100 ns with a 4 fs time step. The temperature and pressure were kept constant at 300 K and 1 atm, respectively, by Langevin coupling. The long-range electrostatic interactions were computed with the Particle–Mesh Ewald method (PME) [85], keeping the cut-off distance of 10 Å to Van der Waals interactions.

### 3.8. Model Validation

The build model of PDE_*Ca* was validated using the PROCHECK software [50,54], ERRAT version 2.0 [55], and Verify 3D [56,57].

### 3.9. Structure Superimposition

The build PDE_*Ca* protein model was aligned to homologous proteins using the PyMOL molecular graphics visualization program [86].

### 3.10. Surface Charge Analysis

Charge and radius calculations were carried out using the PDB2PQR server program [88]. The surface and charge were then visualized in ABPS Tools from the PyMOL molecular graphics visualization program [86].

## 4. Conclusions

In conclusion, a sequence and structural analysis of PDE_*Ca* was carried out using various computational biology programs. The amino acid sequence comparison analysis indicated that SVPDEs display high sequence identity (90.6%) with one another and comparatively low sequence identity (58.33%) with mammalian and bacterial PDEs. The three-dimensional model of PDE_*Ca,* produced using various modeling programs, was of good quality, as shown by the PROCHECK and ERRAT analysis. The modeled structure was further analyzed by molecular dynamic simulation, and the analysis indicated that all important structural parameters, such as chirality, disulfide bonds, and the absence of steric clashes, were correct. The root mean square deviation and radius of the gyration did not suffer significantly during model building and were maintained at 1 Å and 31.7 Å, respectively. The structural analysis indicated that the complex structure of PDE_*Ca* is folded into a multi-domain protein that comprises four domains—a somatomedin B domain, a somatomedin B-like domain, an Ectonucleotide pyrophosphatase/Phosphodiesterase domain (also called autotaxin), and a DNA/RNA non-specific domain. Structural comparisons with PDEs from other snake venoms, and mammalian and bacterial counterparts indicated that the surface charge distribution and the average active site cavity volume and depth vary considerably, which may contribute to their variable substrate specificity. Finally, during the maturation process, venom PDEs lose their signal and activation peptides to convert into the fully active mature forms. The structure of PDE_Cα presented in this paper is only a predicted structure. These conclusions need to be confirmed with experimental evidence [89].

## Figures and Tables

**Table 1:** Percent sequence identities among SVPDEs and their mammalian counterparts.

Proteins	PDE_*Ca*	PDE_*Pm*	PDE_*Ml*	PDE_*Pe*	PDE_*Oo*	5GZ4	6CO1	4B56	5IJQ
PDE_*Ca*	----	85%	93%	92%	96%	85%	64%	50%	47%
PDE_*Pm*	85%	-----	92%	94%	95%	84%	63%	50%	48%
PDE_*Ml*	93%	92%	-----	87%	90%	86%	63%	50%	46%
PDE_*Pe*	92%	94%	87%	------	95%	85%	64%	50%	49%
PDE_*Oo*	96%	95%	90%	95%	------	85%	64%	50%	48%
5GZ4	85%	84%	86%	85%	85%	-----	64%	50%	47%
6CO1	64%	63%	63%	64%	64%	64%	-----	53%	46%
4B56	50%	50%	50%	50%	50%	50%	53%	-----	44%
5IJQ	47%	48%	46%	49%	48%	47%	46%	44%	-----

PDE_Ca: Phosphodiesterase Crotalus adamanteus, PDE_Pm: Phosphodiesterase Protobothrops mucrosquamatus, PDE_Ml: Phosphodiesterase Macrovipera lebetina, PDE_Pe: Phosphodiesterase Protobothrops elegans, PDE_Oo: Phosphodiesterase Ovophis okinavensis, 5GZ4: Phosphodiesterase Naja atra atra, 6C01: human Ectonucleotide pyrophosphatase / phosphodiesterase 3 (ENPP3), 4B56: Ectonucleotide pyrophosphatase phosphodiesterase-1 (NPP1) (Mus musculus), 5IJQ: human Autotaxin (ENPP2).

**Table 2:** Cysteine residues participating in disulfide bridges.

1st Cysteine	2nd Cysteine
34	51
38	75
49	62
55	61
84	101
89	119
99	112
105	111
130	176
138	350
366	466
415	819
555	618
569	675
571	660
767	777

**Table 3:** Salt bridges in the PDE_*Ca* 3D structure.

Residue 1	Residue 2	Distance
NH1 ARG A 58	OD1 ASP A 52	3.59
NH1 ARG A 58	OD2 ASP A 52	2.60
NH2 ARG A 58	OD1 ASP A 52	2.69
NH2 ARG A 58	OD2 ASP A 52	3.30
NH2 ARG A 82	OE1 GLU A 85	3.39
NH1 ARG A 87	OD1 ASP A 104	2.58
NH1 ARG A 87	OD2 ASP A 104	3.54
NH2 ARG A 87	OD1 ASP A 98	2.84
NH2 ARG A 87	OD2 ASP A 98	3.93
NH2 ARG A 87	OD1 ASP A 104	3.31
NH2 ARG A 87	OD2 ASP A 104	2.62
NZ LYS A 102	OD2 ASP A 98	2.69
NZ LYS A 168	OD2 ASP A 158	2.87
ND1 HIS A 189	OD2 ASP A 352	3.42
NE2 HIS A 189	OD2 ASP A 352	3.82
NH1 ARG A 278	OE1 GLU A 302	2.85
NH2 ARG A 278	OE1 GLU A 302	2.88
NE2 HIS A 309	OD1 ASP A 305	2.89
NE2 HIS A 309	OD2 ASP A 305	2.95
NZ LYS A 337	OD2 ASP A 122	3.84
NH2 ARG A 339	OD1 ASP A 287	3.88
NH2 ARG A 339	OD2 ASP A 287	2.58
NE2 HIS A 353	OD1 ASP A 147	3.04
NE2 HIS A 353	OD1 ASP A 352	3.46
NE2 HIS A 353	OD2 ASP A 352	2.96
NH1 ARG A 384	OD2 ASP A 205	2.62
NH2 ARG A 384	OD2 ASP A 205	3.69
NH2 ARG A 384	OD2 ASP A 436	3.73
NZ LYS A 425	OD2 ASP A 727	2.56
NH1 ARG A 426	OD1 ASP A 465	3.74
NH1 ARG A 426	OE1 GLU A 467	3.98
NH2 ARG A 426	OD1 ASP A 465	2.70
NH2 ARG A 426	OD2 ASP A 465	3.11
NH2 ARG A 426	OE1 GLU A 467	3.26
NE2 HIS A 428	OD1 ASP A 816	2.79
NH2 ARG A 434	OD2 ASP A 210	2.94
NE2 HIS A 462	OD1 ASP A 305	3.83
NE2 HIS A 462	OD2 ASP A 305	2.68
NZ LYS A 469	OE1 GLU A 155	3.97
NE2 HIS A 515	OD1 ASP A 502	2.80
NE2 HIS A 515	OD2 ASP A 502	2.97
NH1 ARG A 588	OE1 GLU A 534	3.19
NH2 ARG A 588	OE1 GLU A 534	2.60
NH2 ARG A 645	OD1 ASP A 643	3.26
NH2 ARG A 645	OD2 ASP A 643	2.59
NZ LYS A 710	OE1 GLU A 804	3.97
NH1 ARG A 786	OE1 GLU A 791	3.76
NH2 ARG A 786	OD1 ASP A 788	3.97
NE2 HIS A 810	OE1 GLU A 791	3.37
NH1 ARG A 813	OE1 GLU A 492	3.24
NH2 ARG A 813	OD1 ASP A 816	2.69
NH1 ARG A 815	OE1 GLU A 818	2.75
NZ LYS A 840	OE1 GLU A 818	3.80

NH1 and NH2: Nitrogen atoms (amino groups) of the arginine side chain, OD1, and OD2: Oxygen atoms of aspartic acid side chains, OE: Oxygen atoms of glutamic acid side chains, NZ: Nitrogen atoms (amino groups) of lysine side chains.

**Table 4:** Average active site cavity volumes and average active site cavity depths of SVPDEs and their mammalian and bacterial counterparts.

Protein	Average Volume (Å3)	Average Depth (Å)
PD_*Ca* model	6608.25	21.39
PD_*Vl* model	3985.03	12.54
6C01	14,690.95	19.13
4B56	3651.33	16.30
2GSN	10,107.28	18.58
4ZG7	11,367.42	17.94

**Table 5:** Root mean square deviation values of PDE_*Ca*, PDE_*Vl*, PDE_*Ba,* and their mammalian counterparts.

Protein	RMSD Value
PDE_Ca aligned 6C01	0.21
PDE_Ca aligned 4B56	0.72
PDE_Ca aligned 4ZG7	0.73
PDE_Ca aligned 2GSN	0.92
PDE_Ca aligned PDE_*Vl*	0.57
PDE_Ca aligned PDE_*Ba*	0.56
PDE_Ca aligned 5GZ4	0.60

## Supplementary Materials

The following are available online at https://www.mdpi.com/2072-6651/11/11/625/s1. Figure S1. Molecular dynamic simulation analysis of PDE_*Ca*; Figure S2. Ramachandran plot of the modeled structures of *Vipera lebentia* phosphodiesterase model; Figure S3. Ramachandran plot of the modeled structures of *Bothrops atrox* phosphodiesterase model; Figure S4. Errors plot for the modeled structure of *Vipera lebentia*. The plot was generated by ERRAT2. The amino acid residues showing errors were shown by black lines; Figure S5. Errors plot for the modeled structure of *Bothrops atrox* model. The plot was generated by ERRAT2. The amino acid residues showing errors were shown by black lines; Figure S6. Phylogenetic relationships of PDEs based on protein sequences according to the neighbor-joining method without distance corrections.
