# Supplementary material for: The Sequence and a Three-Dimensional Structural Analysis Reveal Substrate Specificity among Snake Venom Phosphodiesterases
Source: Toxins (Basel). 2019 Oct 28;11(11):625. doi: 10.3390/toxins11110625 (PMC6891707; doi:10.3390/toxins11110625)
Supplement: Supplementary file 1 [file toxins-11-00625-s001.pdf]

Supplementary Materials: The Sequence and a Three-Dimensional Structural Analysis Reveal Substrate Specificity Among Snake Venom Phosphodiesterases

Anwar Ullah, Kifayat Ullah, Hamid Ali, Christian Betzel and Shafiq ur Rehman

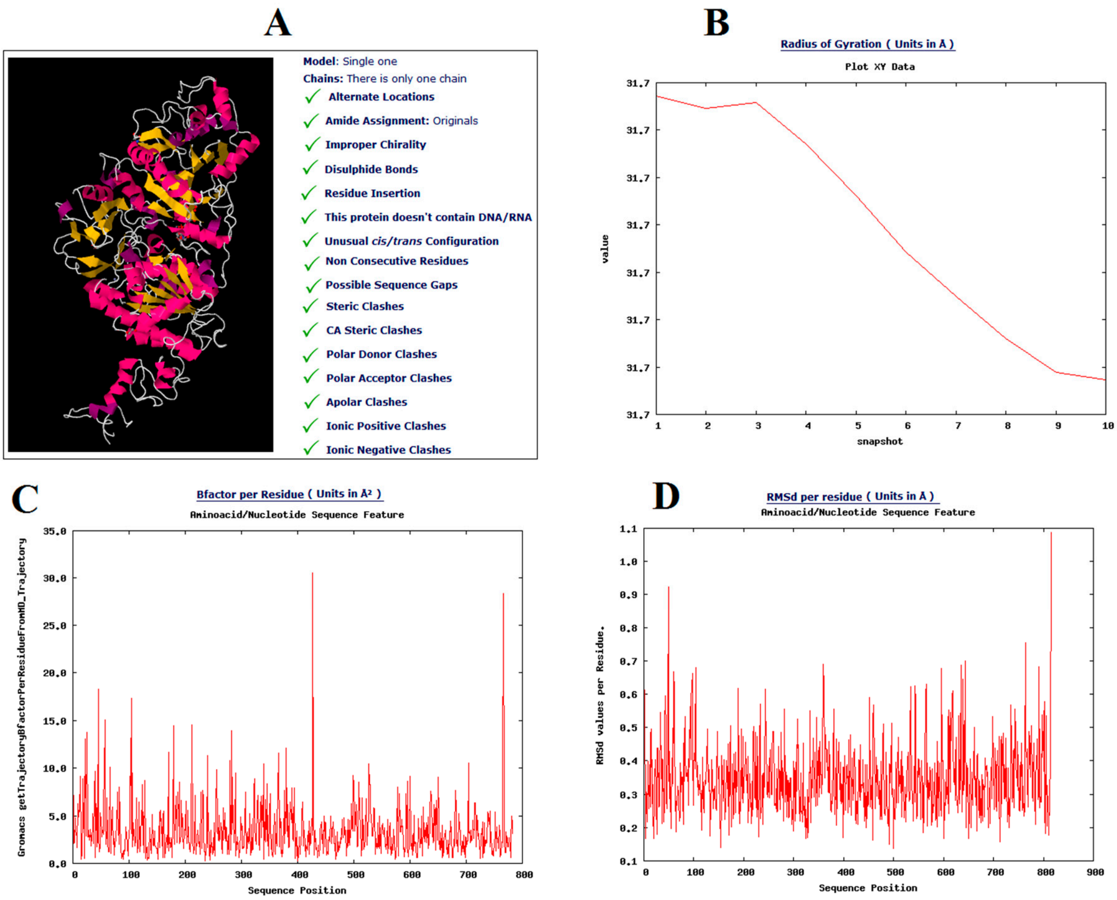

Figure S1. Molecular dynamic simulation analysis of PDE_*Ca.* **(A)** Structural parameters; **(B)** Radius of Gyration; **(C)** B-Factor per residue; **(D)** RMSD per residue.

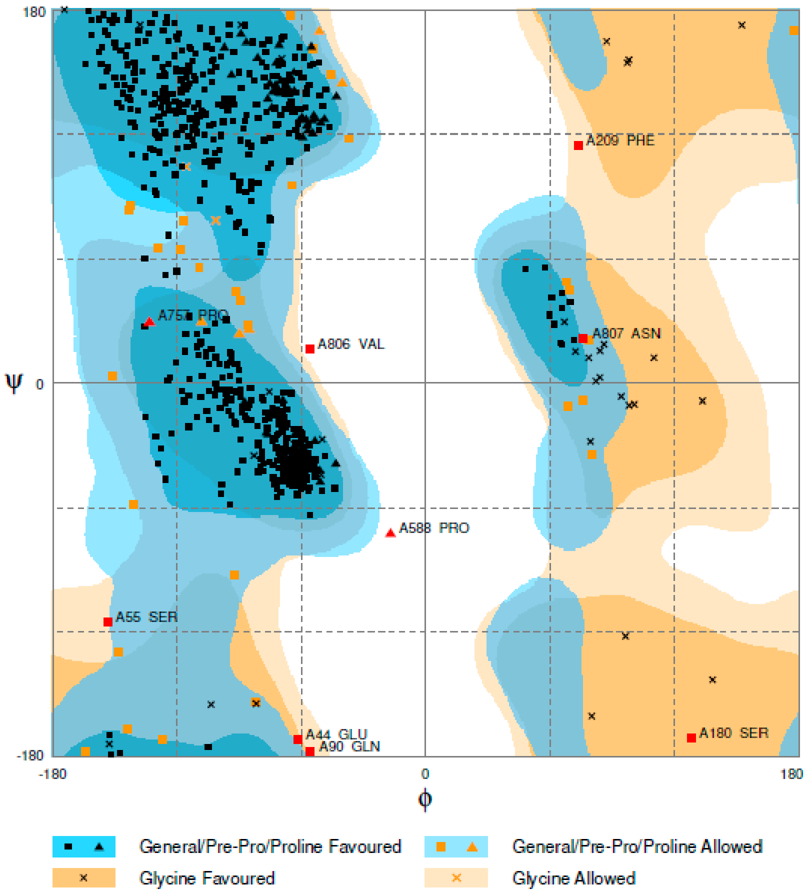

Figure S2. Ramachandran plot of the modeled structures of *Vipera lebentia* phosphodiesterase model.

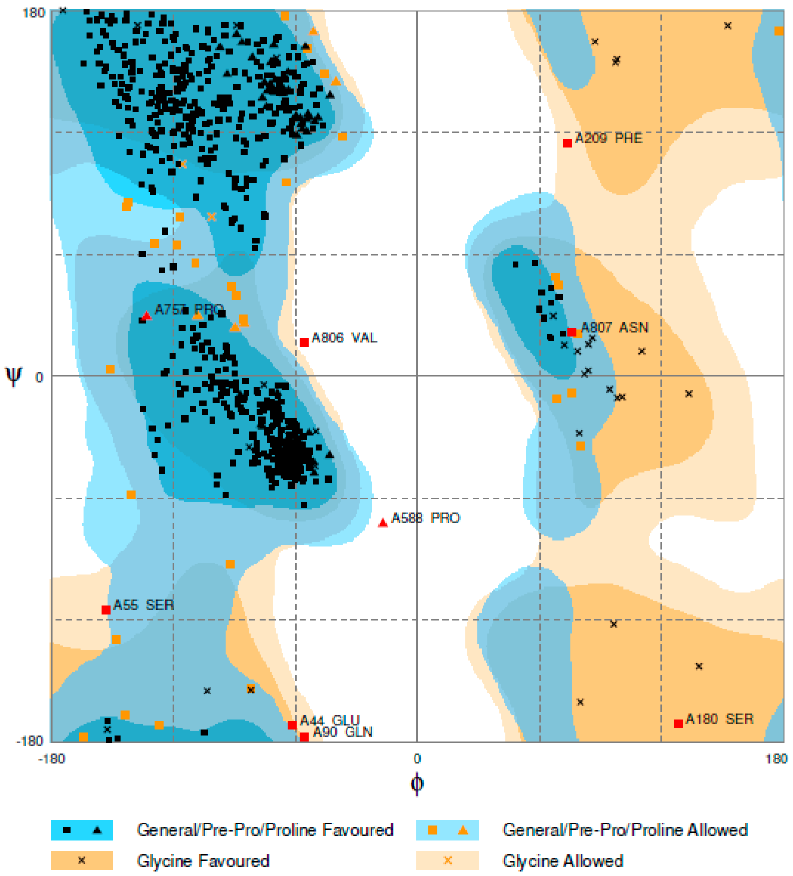

Figure S3. Ramachandran plot of the modeled structures of *Bothrops atrox* phosphodiesterase model.

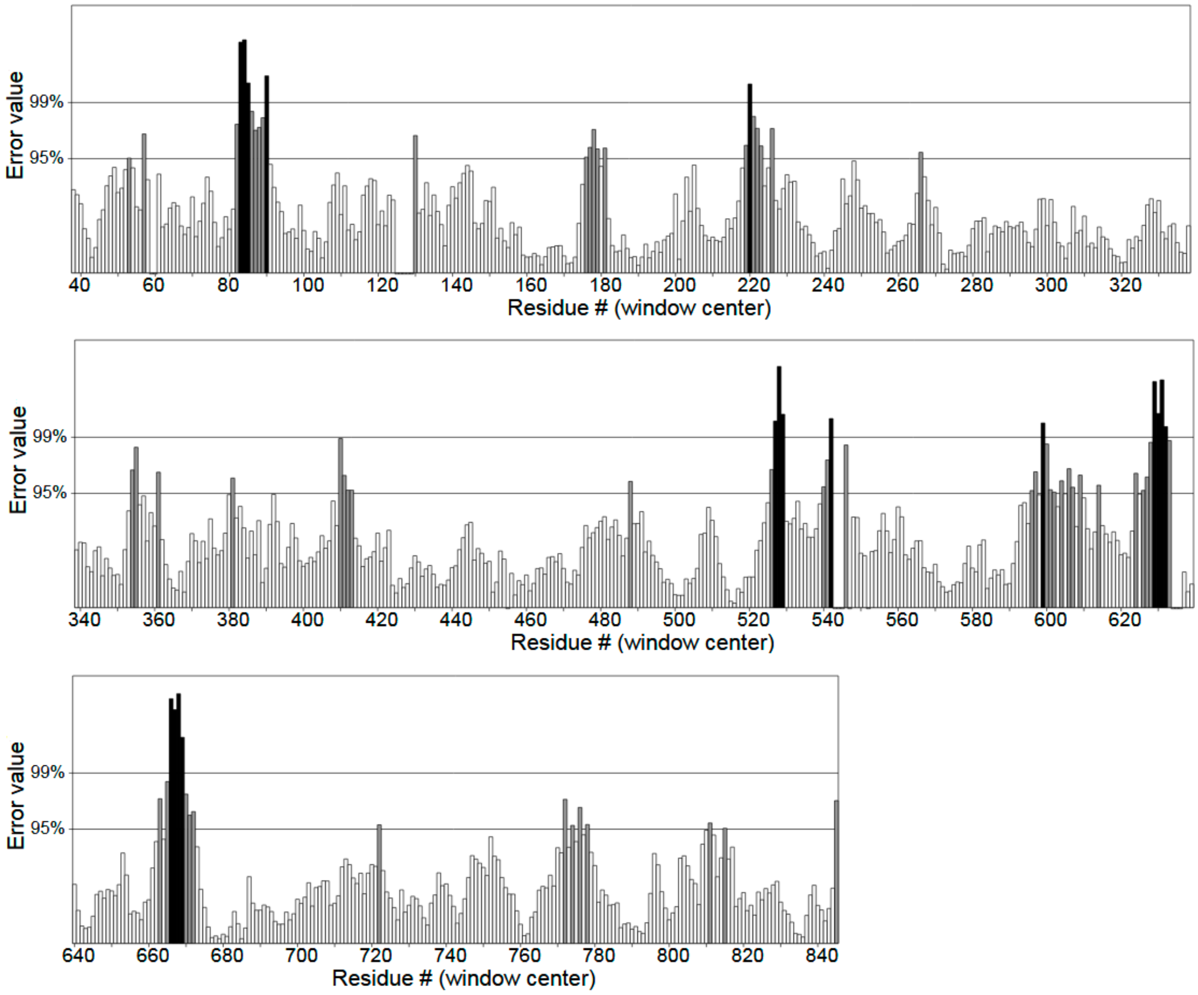

Figure S4. Errors plot for the modeled structure of *Vipera lebentia*. The plot was generated by ERRAT2. The amino acid residues showing errors were shown by black lines.

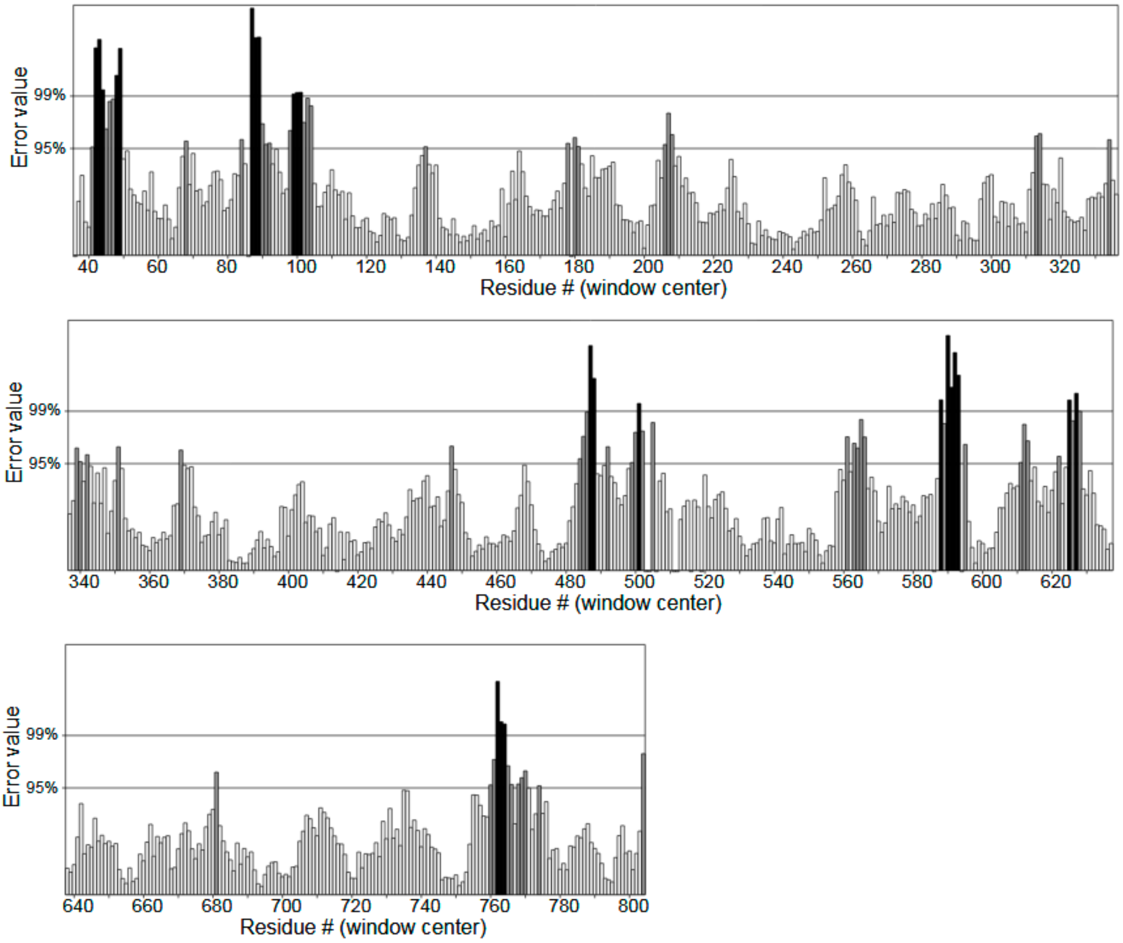

Figure S5. Errors plot for the modeled structure of *Bothrops atrox* model. The plot was generated by ERRAT2. The amino acid residues showing errors were shown by black lines.

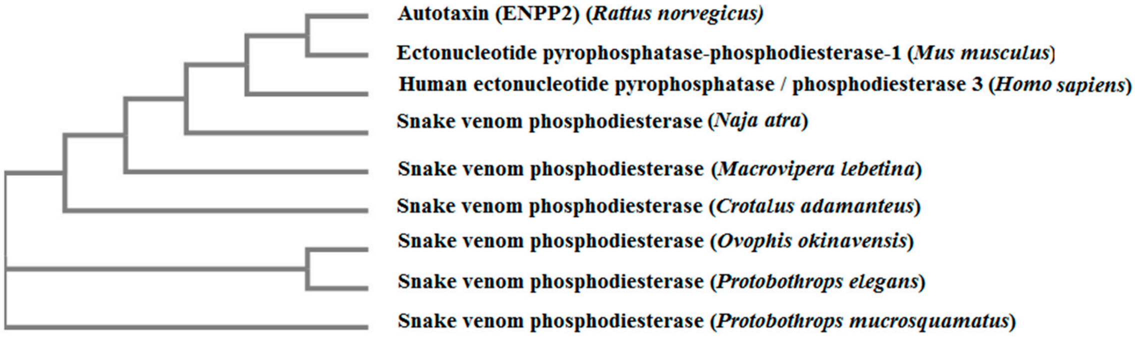

Figure S6. Phylogenetic relationships of PDEs based on protein sequences according to the neighbor-joining method without distance corrections.
